# Supplementary material for: Bond percolation in coloured and multiplex networks
Source: Nat Commun. 2019 Jan 24;10:404. doi: 10.1038/s41467-018-08009-9 (PMC6345799; doi:10.1038/s41467-018-08009-9)
Supplement: Supplementary file 1 — Supplementary Information [file 41467_2018_8009_MOESM1_ESM.pdf]

Supplementary Information for:
Bond percolation in coloured and multiplex networks

Ivan Kryven

[1] *Van 't Hoff Institute for Molecular Sciences, University of Amsterdam, Science Park 904, 1098 XH Amsterdam, The Netherlands*

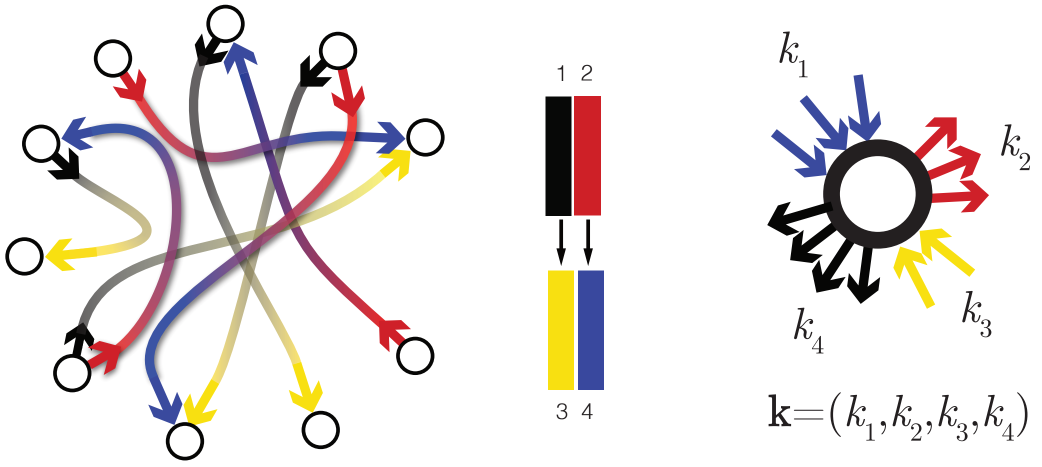

Supplementary Figure 1. Configuration model with coloured directed edges.
In this model, the corresponding pairs of colours are joined according to the matching table.

Mixed nodes	Colour fractions in finite components	Colour fractions in the giant component	Sample of a generated network
0.03	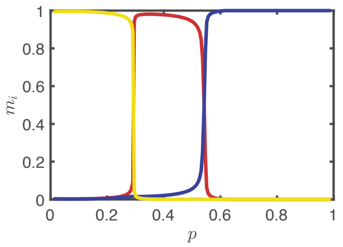	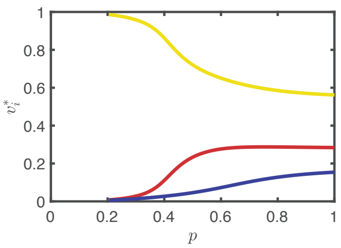	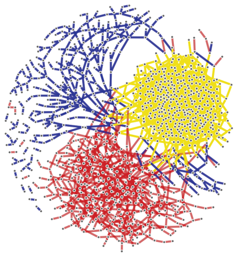
0.15	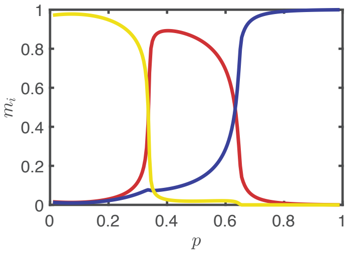	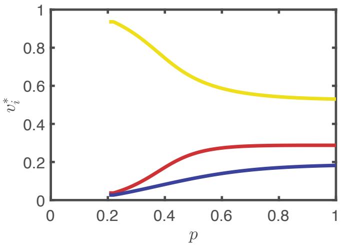	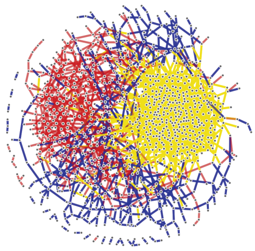
0.3	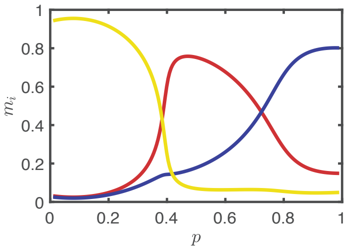	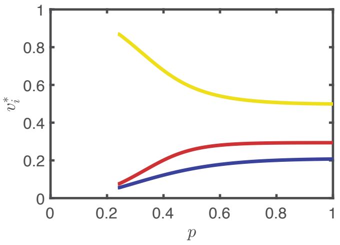	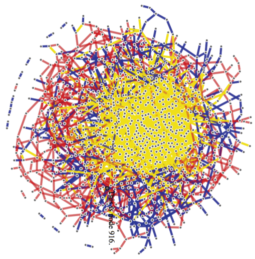
0.8	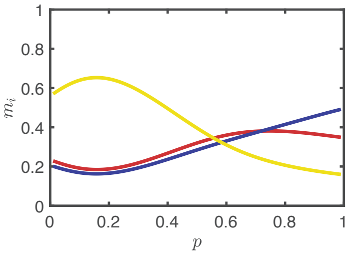	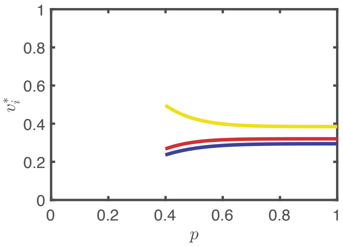	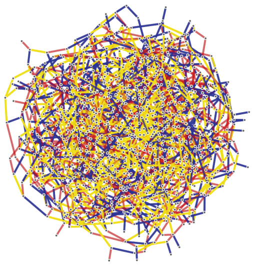

Supplementary Table 1. Parametric study of the colour-switching phenomenon. The fractions of coloured edges in finite and giant components is studied for various fractions of mixed nodes (the nodes having edges of all colours)

Supplementary Note 1: On the interpretation and choice of the edge colouring scheme

Here we provide practical guidelines that allow one to render various classes of networks as essentially the edge-coloured networks.

Networks with multiedges. Let us first discuss how multigraphs can be implemented. A known problem here is that when a network is defined by its degree distribution, the probability to have multi-edges (also known as parallel edges) vanishes in large configuration models. In order to obtain a model in which multiedges do not disappear when the size of the network tends to infinity, one may encode multiedges by single coloured edges, where the colour corresponds to edge multiplicity. Consequently, we obtain N colours in total, where N is the maximum multiplicity. In this way a configuration of a node is encoded by $\mathbf{x} = (x_1, x_2, \ldots, x_N)$, where $x_i \in \mathbb{N}$ denotes the number of edges of multiplicity i. In order to apply the percolation theory, one has to describe how random removal of edges affects such an edge-coloured network. Note that one step of percolation process will only reduce the multiplicity (change colour) of an edge if its multiplicity is greater then one, or remove the edge completely, if the multiplicity of this edge is one. The following master equation implements this logic with the unit rate:

$$\begin{aligned}\frac{\partial}{\partial} u(\mathbf{x}, t) =&(x_1 + 1)u(\mathbf{x} + \mathbf{e}_1, t) - x_1 u(\mathbf{x}, t) + \\ &\sum_{i=1}^{N-1} (i+1)x_{i+1} u(\mathbf{x} - \mathbf{e}_i + \mathbf{e}_{i+1}, t) - \sum_{i=2}^{N} i x_i u(\mathbf{x}, t),\end{aligned} \tag{1}$$

where $\mathbf{e}_i$ form the standard basis, and $u(\mathbf{x}) := 0$ when $\mathbf{x}$ contains negative values. The first line in the right hand side of Supplementary Equation 1 implements the production and consumption of edges of multiplicity one, and the second line – the production and consumption of edges of multiplicity greater then one. The edge-coloured degree distribution $u(\mathbf{x}, t)$ provides the input information for the asymptotic theory of this paper.

Multiplex networks with overlap. Similar logic also applies in the case of edge overlap in multiplex networks[1]. In this case, for each combination of parallel edges one attributes an extra colour that uniquely identifies this combination. In the most general case, there can be at most $2^N - N - 1$ different combinations, each of which has to be encoded with an extra colour, so that one obtains at most $2^N - 1$ colours in total, and therefore, a multivariate degree distribution of corresponding dimensionality.

Directed networks. Consider now an edge-coloured graph in which some edges have a direction. In order to recast this problem in terms of our formalism we have to change the

rules for joining half-edges as used in the definition of the problem, see Supplementary Figure 1. In this framework, we join half edge of colour i only with the corresponding to it colour j where i and j may not be the same. Such colour correspondence is fixed by the permutation matrix $\boldsymbol{P}$: a nonzero element $P_{i,j} = 1$ indicates that all directed edges that start as i^{th} half edge, should end as j^{th} half edge. The following examples of permutation matrices,

$$\boldsymbol{P}_1 = \begin{bmatrix} 0 & 1 \\ 1 & 0 \end{bmatrix}, \ \boldsymbol{P}_2 = \begin{bmatrix} 0 & 0 & 1 \\ 0 & 1 & 0 \\ 1 & 0 & 0 \end{bmatrix}, \ \boldsymbol{P}_3 = \begin{bmatrix} 1 & 0 & 0 \\ 0 & 1 & 0 \\ 0 & 0 & 1 \end{bmatrix}, \tag{2}$$

define correspondingly: one type of directed edges, a mixture of directed and undirected edges (for example as discussed in ref.[2]), and three types of undirected edges. When $\boldsymbol{P} = \boldsymbol{I}$, the model degenerates to the undirected case. Note that the first moments of the degree distribution have to be invariant under the permutation: $\mu_0 = \boldsymbol{P}\mu_0$. The other two important features is that this matrix is symmetric, $\boldsymbol{P} = \boldsymbol{P}^\top$, and involutory, $\boldsymbol{P}^2 = \boldsymbol{I}$. In order to implement this permutation matrix into the theory one has to rephrase the criticality criterion:

$$\mathbf{v} \in \ker[\boldsymbol{M} - \boldsymbol{P}], \ \text{and} \ \frac{\mathbf{v}}{|\mathbf{v}|} > 0, \tag{3}$$

or alternatively, since $\boldsymbol{P}^2 = \boldsymbol{I}$, one can rewrite the latter equation as:

$$\mathbf{v} \in \ker[\boldsymbol{M}\boldsymbol{P} - \boldsymbol{I}], \ \text{and} \ \frac{\mathbf{v}}{|\mathbf{v}|} > 0, \tag{4}$$

where, as before, $|\mathbf{v}| := \sum_{i=1}^{N} v_i$. Note that Supplementary Equation 4 is again an eigenvalue problem.

Node-labelled networks. Finally, consider a network in which all *nodes* are labelled with numbers $1, \ldots, K$, and the edges are unicoloured. One may nevertheless view this network, as being edge coloured where each edge has a *vector* colour (j, k): j and k are the labels of incident to the edge nodes. One can then enumerate this vector colours with a linear index by the following mapping: $(j, k) \to (j-1)K + k$. Since vector colours feature a direction, that is (j, k) corresponds to (k, j), we have to define the permutation matrix by setting: $P_{(j-1)K+k,(k-1)K+j} = 1$ for $k, j = 1, 2, \ldots, K$. We thus formulated the node-labelled network with K labels as a directed, edge-coloured network with K^2 colours.

Supplementary References

1. Cellai, D., López, E., Zhou, J., Gleeson, J. P. & Bianconi, G. Percolation in multiplex networks with overlap. *Phys. Rev. E* **88**, 052811 (2013).

2. Boguná, M. & Serrano, M. Á. Generalized percolation in random directed networks. *Phys. Rev. E* **72**, 016106 (2005).
